# An immuno-score signature of tumor immune microenvironment predicts clinical outcomes in locally advanced rectal cancer

**DOI:** 10.3389/fonc.2022.993726

**Published:** 2022-09-29

**Authors:** Zhengfa Xue, Shuxin Yang, Yun Luo, Ming He, Huimin Qiao, Wei Peng, Suxin Tong, Guini Hong, You Guo

**Affiliations:** ^1^ Medical Big Data and Bioinformatics Research Centre, First Affiliated Hospital of Gannan Medical University, Ganzhou, China; ^2^ Department of Computer Science and Technology, School of Electronic and Information Engineering, Xi’an Jiao Tong University, Xi’an, China; ^3^ School of Information Engineering, Jiangxi University of Science and Technology, Ganzhou, China; ^4^ School of Medical Information Engineering, Gannan Medical University, Ganzhou, China

**Keywords:** immuno-score signature, locally advanced rectal cancer, neoadjuvant chemoradiotherapy, immune gene pairs, prognostic prediction

## Abstract

**Background and purpose:**

Accumulating evidence indicates that neoadjuvant chemoradiotherapy(nCRT) success has an immune-associated constituent in locally advanced rectal cancer (LARC). The immune-associated configuration of the tumor microenvironment associated with responses to treatment was explored in LARC in this study.

**Material and methods:**

A novel analytic framework was developed based on within-sample relative expression orderings for identifying tumor immune-associated gene pairs and identified an immuno-score signature from bulk transcriptome profiling analysis of 200 LARC patients. And sequencing and microarray analysis of gene expression was conducted to investigate the association between the signature and response to nCRT, immunotherapy, and cell function of CD4 and CD8. The results were validated using 111 pretreated samples from publicly available datasets in multiple aspects and survival analyses.

**Results:**

The immuno-score signature of 18 immune-related gene pairs (referred to as IPS) was validated on bulk microarray and RNA-Seq data. According to the model’s immune score, LARC patients were divided into high- and low-score groups. The patients with high-score were greater sensitivity to nCRT and immunotherapy, gaining a significantly improved prognosis. In addition, the immune-score gene pair signature was associated with type I anti-tumor T cell responses, positive regulators of T cell functions, and chromosomal instability while reflecting differences between CD8+ T cell subtypes.

**Conclusion:**

The immuno-score signature underlines a key role of tumor immune components in nCRT response, and predicts the prognosis of LARC patients as well.

## Introduction

The clinical diagnosis and treatment of locally advanced rectal cancer (LARC) are extremely challenging. Despite great efforts have been made for systematic diagnosis and surgical intervention, clinical outcomes remain varied drastically. Growing evidence suggests that the immuno-associated microenvironment plays an important role in tumor behavior (1, 2). Enhancing efficacy of preoperative neoadjuvant chemoradiation(nCRT) and nivolumab for LARC subtypes suggests that immunological analysis of tumor immune scores has shown great potential for predictive diagnosis, prognosis, and response to immunotherapy in locally advanced rectal cancer patients (LARCs) (3). Indeed, immune-infiltrating plays a critical role in the tumor microenvironment and has been involved in the treatment of most cancers (4–6). However, the roles of nCRT-immune interactions of LARCs remain largely unknown. Therefore, there is an urgent need to explore effective and novel immune-grade signatures in LARCs, to reveal resistance mechanisms and to develop potential tools for enhancing treatment efficacy.

Recently, a growing number of immune analyses based on gene expression profiles are used to predict the prognosis of LARCs (7–10). However, comprehensive analysis of bulk data remains challenging due to technical design differences, platform variations, and batch effects (11). Our previous evidence proved that the within-sample relative expression orderings (REO) of gene pairs can robustly resist batch effects (12–14). Given the nCRT response can be shaped by intra-tumoral immune constituents, and the advantage of REO analysis, this study focused on the immuno-related gene pairs (IRGPs) derived from bulk data, and to identify an IRGPs-based predictive signature for personalized treatment of LARCs.

In this study, immuno-related gene pairs associated with responses to nCRT were assessed in pretherapy biopsies from LARCs, and an immuno-score signature based on IRGPs was constructed for prognostic prediction. Furthermore, the immune score signature differentiated LARCs who responded to immunotherapy, which may develop a more effective treatment for novel immunotherapy strategy.

## Materials and methods

### Data source and pre-processing

All transcriptome expression profiles and clinical information of LARCs and validation cohorts in this study were obtained from the Gene Expression Omnibus repository (GEO). The *immport* database (https://www.immport.org/) offered a total of 1245 immune-related genes. In GSE39582 (15), 585 samples with clinical information were measured using the Affymetrix Human Genome U133 Plus 2.0 Array (GPL570) platform. We selected 200 LARCs who received nCRT as the training dataset. The gene expression profiles and its clinical characteristics of 332 patients from the GEO database were used to validate our signature.

The samples in cohorts GSE87211 (16), GSE35452 (17) and GSE45404 (18) were all patients who received nCRT. They were used to validate the effectiveness of the immuno-score signature about the pathological response status and prognosis of LARCs. Details of validation cohorts in this study are shown in **Table 1**.

**Table 1:** Datasets analyzed in this study.

GEO ID	Platform	Sample size	Tissue sample type	Reference
GSE39582	GPL570	200	LARC	(15)
GSE87211	GPL13497	111	LARC	(16)
GSE35452	GPL570	46	LARC	(17)
GSE45404	GPL570	42	LARC	(18)
GSE100109*	GPL23593	10	Rectal cancer	(19)
GSE99897*	GPL11154	10	Rectal cancer	(19)
GSE119409	GPL570	66	Rectal cancer	(7)
GSE113585	GPL20301	34	Colorectal cancer	(20)
GSE34489	GPL570	33	Colorectal cancer	(21)

* The two-expression profiling are array-based and sequence-based homology data, respectively.

To investigate whether the immune scores of IRGPs-based signatures were consistent on array-based and sequence-based data, we collected 10 cases (19) with two types (array-based and sequence-based) of expression profiles to verify the consistency of the prediction results of signature. To further investigate the correlation between immuno-score signature and response to nCRT, we divided 66 patients from GSE119409 (7) into sensitive and resistant groups based on mRNA expression in pre-therapy biopsies and compared the immune score of the two groups. To explore the relationship between LARC and the immune-infiltrating microenvironment, we further investigate the relationship between immune score and CD8 cell expression (GSE113585) (20), chromosome instability phenotype (GSE34489) (21), T cell positive regulator genes.

### Establishment of the immuno-score signature

**Figure 1:**
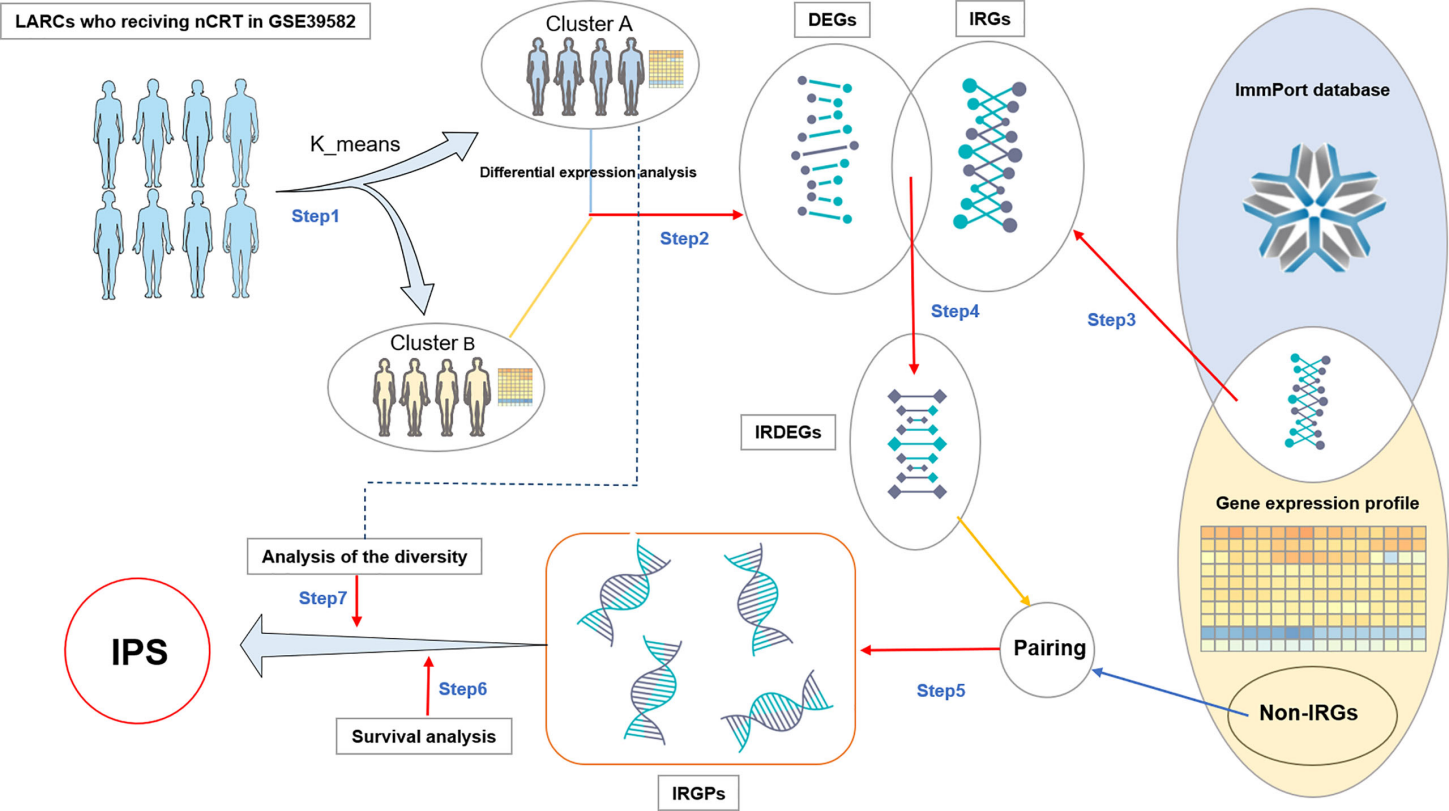
Flowchart of IPS discovery process. DEGs: differentially expressed genes. IRGs: Immune-related genes. IRDEGs: Immune-related differentially expressed genes. Non-IRGs, Non-immune-related genes; IRGPs, Immune-related gene pairs; IRGPS, Immune-related gene pair Signature.

As shown in **Figure 1**, the construction process of the immuno-score signature is divided into the following seven steps:

I. We divided LARCs from the GSE39582 cohort into two groups, cluster *A* and cluster *B*, by using the *k-means* clustering algorithm with the expression value of the gene as a factor (22). The distance between two samples is calculated using the Euclidean distance
II. We searched for significantly differentially expressed genes (DEGs) between cluster *A* and cluster *B* by differential expression analysis (*FDR* < 0.05).
III. Based on the list of immune-related genes (IRGs) in the *immport* database, we extracted IRGs from the GSE39582 cohort.
IV. These immune genes were intersected with DEGs to obtain immune genes with significant differential expression, which were recorded as IRDEGs.
V. We paired the IRDEGs with all other genes (non-immune genes and immune genes) in the GSE39582 expression profile to obtain IRGPs. The expression values of the two genes in the gene pair were recorded as *Ga* and *Gb* respectively. If *Ga > Gb*, the score of the gene pair was recorded as 1 score (1s), otherwise 0 score(0s).
VI. Patients were divided into two groups according to the score (0s or 1s) of each gene pair in IRGP, and we screened for patients with significant differences in survival between groups (*P* < 0.05). These gene pairs were marked as candidate gene pairs.
VII. Finally, we define a diversity factor *α* to identify the final gene pairs to form an immuno-score signature. *α* is a decimal in the interval [0,1]. For each gene pair, the diversity factor α = 0.6 means the score of 60% or more of the samples in cluster *A* were 1s, while the score of 60% or more of the samples in cluster *B* were the 0s (*A* and *B* reverse also applicable). The final retained gene pairs would be used to form an immuno-score signature.

### Assessment of immune microenvironment

Immunophenoscore (IS) and tumor purity score(TPS) were assessed by utilizing the R package *hacksig* (version 0.1.2) (23, 24) to GEO gene expression data.

### Functional enrichment analysis

The gene ontology enrichment analysis was performed on the online platform *DAVID* Bioinformatics Resources 6.8 (https://david.ncifcrf.gov/), and the R package *Goplot* was used for drawing. Enriched pathways with FDR less than 0.05 were considered statistically significant.

### Independence of the immuno-score signature from other LARC patients’ clinical characteristics

To examine whether the immuno-score-based prognostic model was an independent variable when considering other conventional clinical features (age, gender, tnm stage) in LARCs, multivariate cox regression analyses were performed.

### Differential expression analysis and survival analysis

The R package *limma* was used to identify differentially expressed genes (*FDR <0.05 and |log FC |>2*). The log-rank test in the software *GraphPad Prism 8* was used to evaluate the survival analysis of the training dataset and validation dataset. In all statistical analyses, *P* < 0.05 was considered statistically significant.

## Result

### Construction of the IPS immune scoring system

As shown in **Figure 1**, firstly, we divided the patients into two groups by unsupervised analysis of the k-means algorithm with the expression value of the gene as a factor (22). There were significant differences in gene expression between the two groups of patients. One group contained 99 patients and the other 101. In step two and three, we got 11456 DEGs and 1257 IRGs. In step four, we obtained 936 IRDEGs by intersecting DEGs with IRGs. In step five, pairing IRDEGs with other genes, we got 18,292,716 IRGPs. Then in step six, we got 16,544 candidate gene pairs. In the last step, to control for the number of gene pairs in the immuno-score signature to be less than 50, we set the diversity factor to 0.8 and finally obtained 18 immune-related gene pairs signature (referred to as IPS; **Table 2**).

**Table 2:** The composition of IPS.

Gene pairs (*G_a_ > G_b_* *)	Gene pairs (*G_a_ > G_b_*)
*1. CXCL11 > ETNK2*	*10. CXCL11 > TSPYL5*
*2. CXCL11 > FZD9*	*11. CXCL11 > DRD4*
*3. CXCL11 > CDH5*	*12. CXCL11 > LOC101928837*
*4. CXCL11 > ZSCAN2*	*13. CXCL10 > SP2*
*5. CXCL11 > PAMR1*	*14. CXCL10 > PLOD1*
*6. CXCL11 > LRP12*	*15. TNFSF4 > CSPG4*
*7. CXCL11 > NEURL2*	*16. TNFRSF10A > CDHR2*
*8. CXCL11 > DAAM2*	*17. GZMB > ACKR3*
*9. CXCL11 > CDK5R1*	*18. NEDD4 > CAMSAP3*

* G_a_ and G_b_ represent expression values of genes. If G_a_ > G_b_, the score of the gene pair was recorded as 1 score, otherwise 0 score.

**Figure 2:**
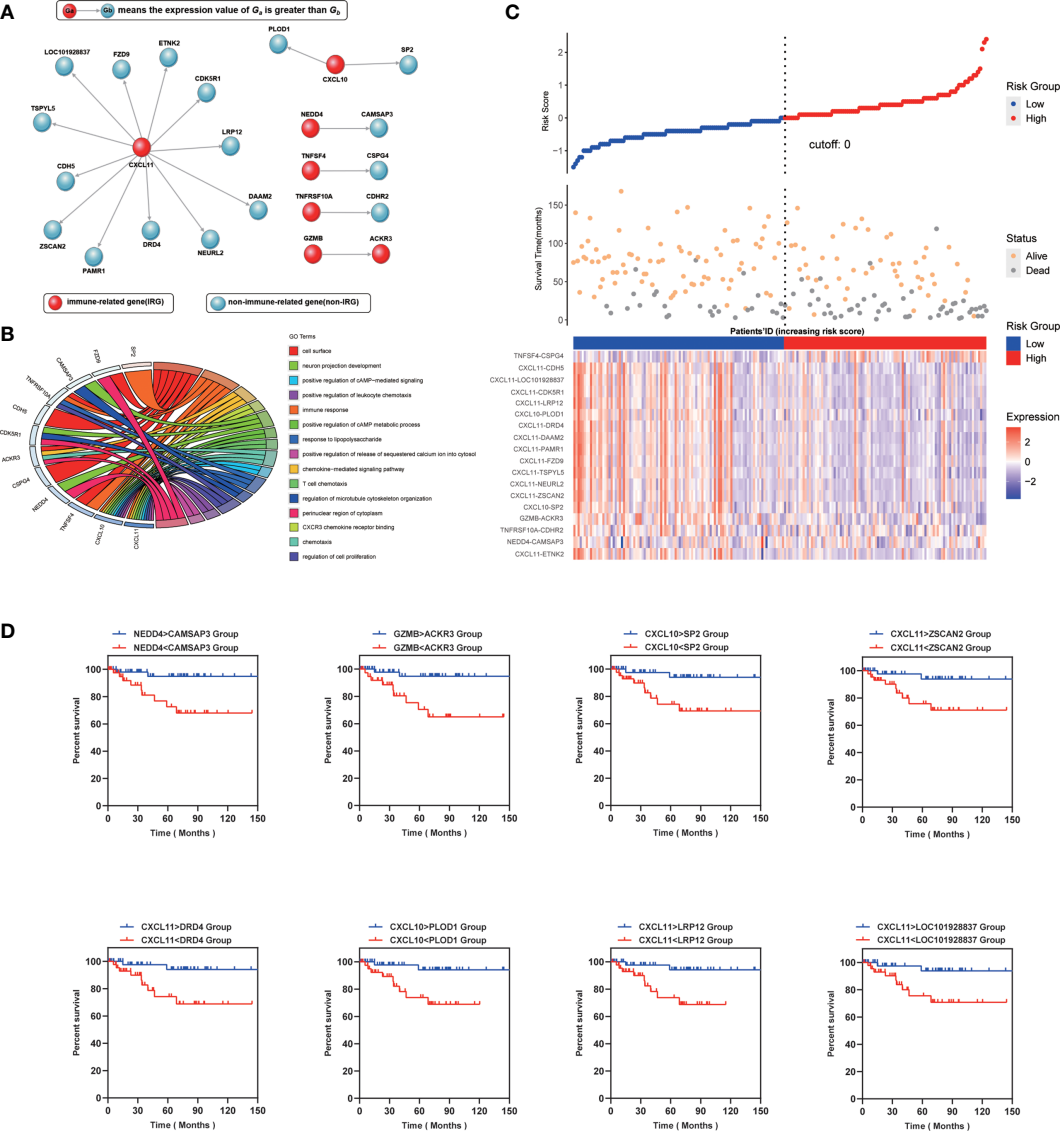
**(A)** Genetic makeup of IPS. **(B)** Enrichment distribution of differentially expressed genes in IPS. Different colors represent different access paths. **(C)** Up: The risk scores obtained by the COX regression of all patients are arranged in order of risk from low to high, and then a scatterplot is made. The X-axis is the serial number of the patient, and the Y-axis is the risk score. The blue dots are the low-risk group, and the red dots are the high-risk group. Middle: The X-axis is the patients’ ID, the Y-axis is the survival time, the orange dots indicate the outcome is survival, and the gray dots indicate the outcome is death. Down: Heatmap of IPS profiles in the high- and low-risk groups. It is calculated as the expression value of the former gene minus the latter. **(D)** Kaplan–Meier curve analysis of eight representative immune-related gene pairs in validation cohort (GSE87211).

IPS contains 24 genes, of which seven genes (CXCL11, CXCL10, NEDD4, TNFSF4, TNFSF4, TNFRSF10A, GZMB) are related to immune function. Other genes are unclear and need to be further revealed **(** **Figure 2A** **)**. To gain further insight into the underlying biological characteristics based on the DEGs between high- and low-risk LARCs, we performed the GO enrichment analysis on the constituent genes of IPS. The result showed that DEGs were most enriched in functions such as cell surface, immune response, and perinuclear region of cytoskeleton organization **(** **Figure 2B** **)**.

Subsequently, a risk score of each LARC patient was calculated, and patients were then separated into low risk and high risk by the COX regression. We found that the results of the COX regression were consistent with the results of the IPS score (**Figure 2C**). We grouped patients by the score of IPS and then compared survival between the two groups **(** **Figure 2D** **)**. Representative Kaplan-Meier plots show that gene pairs associated with CXCL10 and CXCL11 were related to prolonged survival in LARCs, which can function independently of IPS. Therefore, we assigned each LARC patient a score (range 1 to 18) by using IPS. Patients were then divided into low- and high-score groups using the median risk score as a cutoff value and tested for significance with a rank-sum test (25).

**Figure 3:**
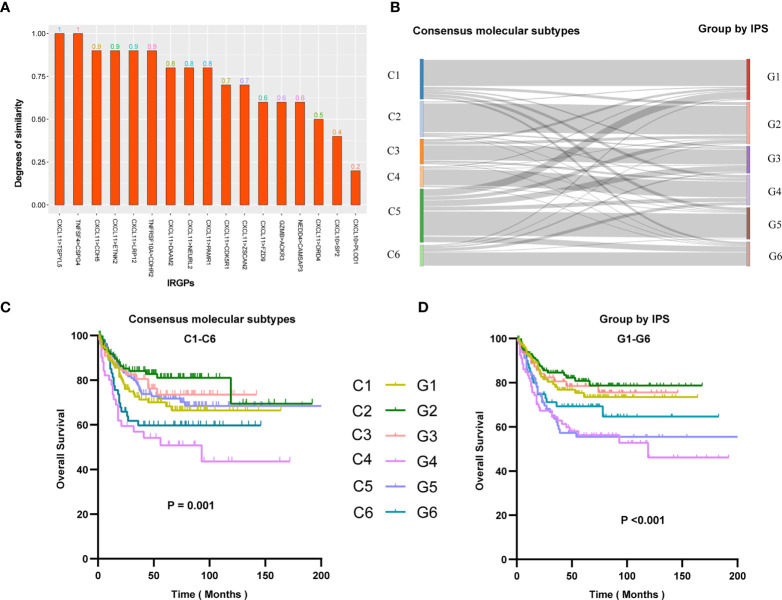
**(A)** Similarity of IPS results in array-based and sequence-based data. Similarity represents the proportion of samples with consistent IPS results in array-based and sequence-based data. The abscissa is the name of the gene pair, and the ordinate is the similarity of the gene pair in the two sets of data. **(B)** Re-stratification of patients from six consensus molecular subtypes to the novel six immune score-based group of IPS. Each line represents a patient. Each line represents a sample. Kaplan–Meier analysis for patients in GSE39582 stratified by consensus molecular subtypes **(C)** or IPS risk stratification system **(D)**.

To ensure cross-platform comparability, we investigate the consistency of the prediction results of IPS in different types of gene expression profiles. We collected two sets of expression profiling data from 10 rectal cancer patients, which are array-based and sequence-based data, respectively. And we calculated the scores (0s or 1s) of gene pairs in all samples and counted the proportion of concordant samples. The results showed that IPS performed well on expression profiling of different technology platforms, with two-thirds of the gene pairs possessing a concordance ratio of more than 0.7 (**Figure 3A**).

To better understand the differences in immune scores between different molecular groups classified by the consensus unsupervised approach (15), we explored the relevance of an IPS-based grouping model with consensus molecular subtypes. The paper of the cohort GSE39582 provided six consensus molecular types (referred to as C1-C6) and the molecular characterization of each of these subtypes. we tried to re-stratify the patients to groups 1-6 (referred to as G1-G6) according to the IPS score. We were surprised to find that the novel stratification can be well mapped to the consensus molecular types **(** **Figure 3B** **)**. Survival analysis showed that the novel stratification can also accurately define the prognosis of patients **(** **Figures 3C, D** **)**. Therefore, the IPS-based stratification system can provide a reference for the definition of novel molecular subtypes in the future.

### Predicts the prognosis and response to neoadjuvant treatment

**Figure 4:**
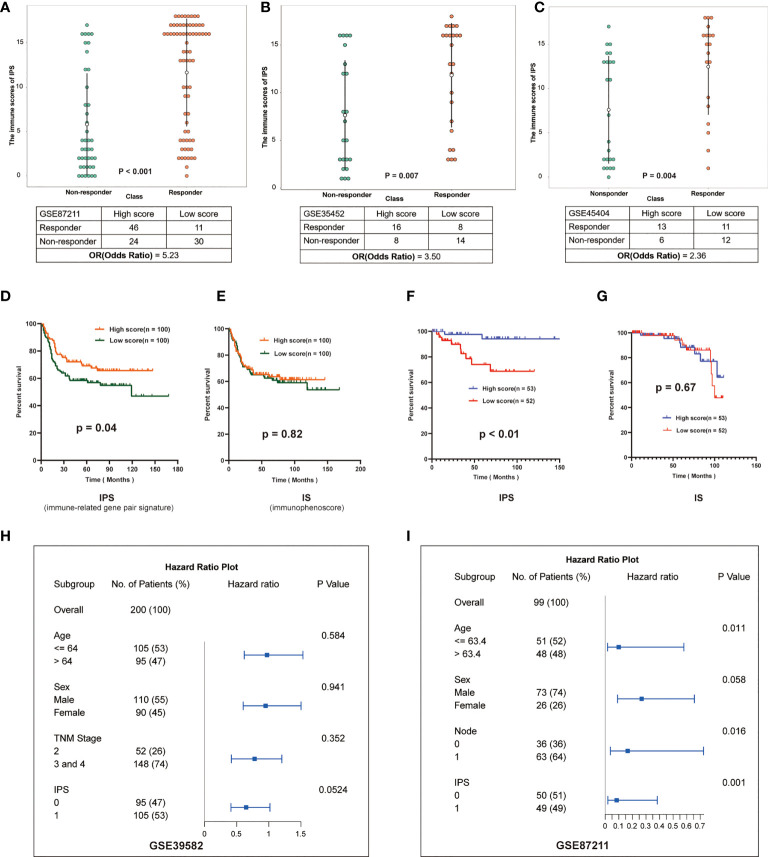
Validation of IPS in multiple datasets. **(A–C)** Distribution of immune scores in different groups of GSE87211, GSE35452 and GSE45404. The patients were grouped according to the response to nCRT. The abscissa represents different patient groups, and the ordinate represents the level of immune scores. **(D, E)** Kaplan-Meier survival curves of the DFS in the high- and low-score groups based on IPS and IS (GSE39582). **(F, G)** Kaplan-Meier survival curves of the DFS in the high- and low-score groups based on IPS and IS (GSE87211). **(H, I)**: Multivariate analysis of GSE39582 and GSE87211, hazard ratio and p-values were calculated using log-rank test. “Node” indicates the lymph node metastasis status of the patient before surgery, 1 indicates metastasis, 0 indicates no.

To investigate impacts of the immune-associated configuration of the tumor microenvironment on cancer neoadjuvant treatment, we applied IPS to LARCs from GSE87211, GSE35452 and GSE45404. According to the scoring rules of IPS, we classified the patients with scores greater than 9 as the high-score group, otherwise the low-score group. In the validation cohort GSE87211, 46 of 57 (80.70%) patients who responded to nCRT were classified as a high-score group, while 30 of 54 (55.56%) non-responders were classified as a low-score group (rank-sum test, p < 0.001), the odds ratio is 5.23 **(** **Figure 4A** **)**. In the second validation cohort GSE35452, 16 of 24 (66.67%) patients who responded to nCRT were classified as a high-score group, while 14 of 22 (63.64%) non-responders were classified as a low-score group (rank-sum test, p = 0.007), the odds ratio is 3.50 **(** **Figure 4B** **)**. In the third verification cohort (GSE45404), 13 of 24 (54.17%) patients who responded to nCRT were classified as a high-score group, while 12 of 18 (66.67%) non-responders were classified as a low-score group (rank-sum test, p = 0.004), the odds ratio is 2.36 **(** **Figure 4C** **)**.

Furthermore, we investigated effects of the immune-associated configuration on patient survival by predicting the LARCs prognosis based on the IPS. Firstly, we calculated tumor purity scores (TPS) for patients in GSE3952 and GSE87211 by the R package *hacksig* (23). In GSE39582, the mean TPS of the high-score group was 0.338, and the low-score group was 0.388. In GSE87211, the mean TPS of the high-score group was 0.251 and the low-score group was 0.296. We found there is no significant difference in mean TPS between the two groups of patients, suggesting no correlation between tumor cell purity and IPS-based immune score in LARCs.

Thus, according to the scoring rules of IPS, patients were divided into a high-score group and a low-score group by the median. Kaplan-Meier analysis showed that low-score LARCs had significantly lower DFS than high-score LARCs (**Figures 4D, F**). In contrast, Kaplan-Meier analysis showed there was no significant difference in survival between two groups based on the median immunophenoscore (IS) (23, 24) (**Figures 4E, G**), suggesting that the score system of immunophenoscore failed to exploit the opportunity of predicting the LARCs prognosis.

Finally, we performed a multivariate analysis of IPS to further investigate whether the prognostic model based on IPS was an independent indicator considering other routine clinical factors. In cohorts GSE39582, cox regression analysis showed that only IPS was associated with patient survival **(** **Figure 4H** **)**. In cohort GSE87211, cox regression analysis showed that age, N stage, and IPS were associated with patient survival, while IPS-based prognostic models were more reliable than other clinical parameters **(** **Figure 4I** **)**. Therefore, IPS can be used as an independent prognostic indicator for LARCs.

### Predicting response to the combination of radiotherapy and suppression of tregs in LARCs

**Figure 5:**
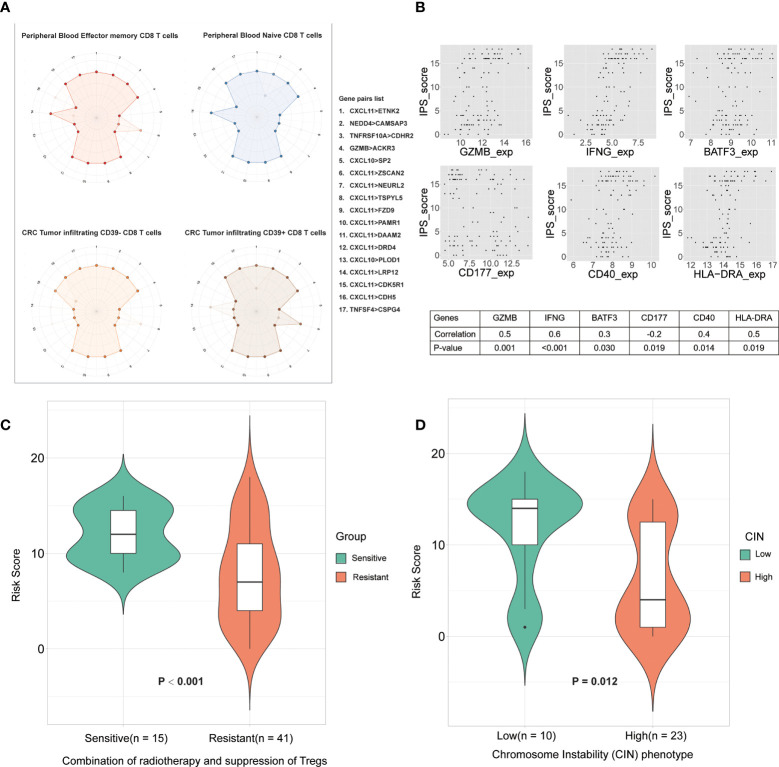
The relationship between IPS and the immune microenvironment. **(A)** Differences in CD8 T cell subtype scoring in IPS. Numbers 1 to17 (one missing) represent different gene pairs. Points in the outer circle are points with a score of 1, and points in the inner circle are points with a score of 0. Finally, overlay the plots for all samples of the same type. **(B)** Correlation between IPS-based immune scores and type I antitumor T cell expression values. The abscissa is the gene expression value, and the ordinate is the score. Finally, the correlation between them was calculated. **(C)** Immune scores between sensitive group and resistant group to radiotherapy. **(D)** Correlation between IPS-based immune score and chromosome instability phenotypes.

To investigate impacts of the immune-associated configuration of the tumor microenvironment on immunotherapy, we applied IPS to a dataset, GSE119409 (7), which comprised 66 LARCs from a clinical trial on anti-Tregs therapy, and investigated the correlation between IPS-based immune scores and response to the combination of radiotherapy and suppression of Tregs. We divided patients into responding and non-responding groups based on IPS, and compared their immune scores. Thus, we found that the IPS of patients who were sensitive to the combination therapy were significantly higher than the non-responders (p < 0.001; **Figure 5C** **)**, which suggested that IPS can provide important insights on the tumor immune infiltration and offer valuable indicator for anti-Tregs therapy response during immunotherapy.

### IPS reflects the difference between the subtypes of CD8+T cells

To explore the heterogeneity of the immune composition and the phenotypic profile of tumor-infiltrating lymphocytes (TILs) within individual tumors and between patients, we calculated differences in scores between different CD8+ T cell subtypes using IPS. We superimposed the scoring maps based on the IPS scoring system of CD8+ T cell samples of the same subtype and found that the IPS-based immune scoring system could distinguish CD8 Tells of different states **(** **Figure 5A** **)**. The results showed that peripheral blood effector and memory T cells differed in scores from peripheral blood naive T cells in some gene pairs (NEDD4>CAMSAP3, CXCL11>ZSCAN2, and CXCL11>LRP12). We also compared the scoring characteristics of CD39–CD8+ and CD39+ CD8+ TILs, and the results showed that there were significant differences in gene pairs such as CXCL11>ZSCAN2, CXCL11>LRP12, etc. Therefore, the IPS-based immune scoring characterized the cell types and states in the immune microenvironment. In addition, the expression frequency of CD39 in CD8+ TILs correlate with the prognosis of patients, which further validated the validity of the IPS-based prognostic model.

### IPS correlates with type I anti-tumor T-cell responses

CD40 expression is positively correlated with type I anti-tumor T-cell responses and better survival (26). To explore the correlation between IPS-based immune scores and type I antitumor T cell (CD4, CD8A, CD40, CD40LG, GZMB, HLA-DRA, IFNG, IL5, PAX5, BATF3, CD177, BCL2) responses, we calculated the correlation of the above gene expression values with IPS scores in the GSE87211 cohorts and plotted the genes that were significantly associated **(** **Figure 5B** **)**. The results showed that the IPS score was significantly correlated with the expression of genes such as CD177 (granulocytes), CD40, BATF3 (dendritic cells), IFNG and GZMB (type I antitumor response) were correlated. Therefore, IPS scores correlated with T-lymphocyte markers and conventional cellular markers, showing great potential in the immunotherapy of colorectal cancer.

### IPS correlates with chromosome instability

The chromosomal instability (CIN) phenotype is a predictive signature and can be used to predict survival for stages II and III of colorectal cancer (21). Hence, we compared the immune scores between the high- and low-CIN phenotype in cohort GSE34489. The results proved that patients with low CIN had significantly higher scores than those with high CIN **(** **Figure 5D** **)**. Both low immune scores and high-CIN phenotype were associated with worse survival, so this result further supports that IPS has maintained a close association with the CIN, as well as clinical outcomes of patients.

### IPS is associated with positive regulators of T cell functions

**Figure 6:**
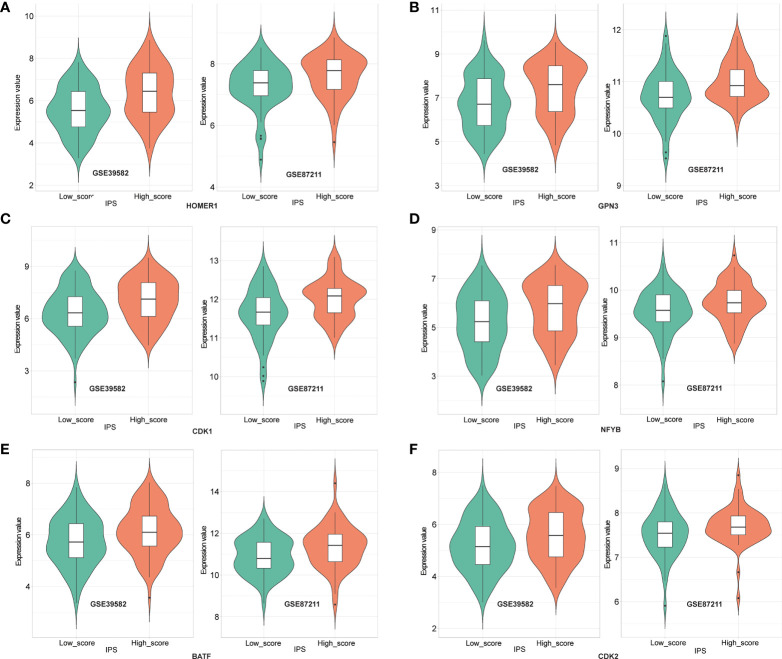
Expression of HOMER1 **(A)**, GPN3 **(B)**, CDK1 **(C)**, NFYB **(D)**, BATF **(E)** and CDK2 **(F)** between high- and low- IPS score groups in GSE39582 (left) and GSE87211(right) (t-test, p<0.05).

To decompose the functional underpinnings of the IPS, we explored the relationship between IPS and top-ranked open reading frames (ORFs), which were identified through overexpression of around 12,000 barcoded human ORFs and associated with the proliferation, activation, and cytokine secretion of CD4^+^ and CD8^+^ T cells (27). First, we used a total of 311 LARCs from GSE39582 and GSE87211 to calculate the correlation between gene expression of the top-ranked ORFs, and genes significantly related to each other were selected for subsequent analysis. We then compared the gene expression between the high IPS score group and the low IPS score group. We found that the expression values of 6 genes (HOMER1, GPN3, CDK1, NFYB, BATF, CDK2) showed significant differences between high- and low-score groups (p < 0.05), and the difference showed consistency in both cohorts **(** **Figure 6** **)**. The results revealed that the IPS-based immune score functions in diverse pathways of relevance to T cell fitness, and showed different modes of endogenous regulation.

## Discussion

In the past, immune factors in the nCRT response of LARCs have been largely ignored. Recently, the presence of pretherapy immune cell infiltration correlated with response to radiotherapy or chemotherapy in LARC has gradually begun to be emphasized (27–29). In consequence, identifying a reliable and feasible scoring system related to the immune microenvironment may play a significant role in the clinical management of LARCs.

In this study, we presented a new approach to data analysis, which combines within-sample relative expression orderings with machine learning algorithms, and obtained a robust predictive signature of the immune-scoring system. These results showed that the signature of 18 immune-related gene pairs natively eliminates the need to consider batch effects caused by different platforms, and accurately predicts the prognosis of LARCs after nCRT. In addition, the IPS showed consistent results on bulk array-data and sequence-data, and it also corresponds to consensus molecular subtypes (15). Of note, no research has yet constructed a prognostic signature of LARC by IRGPs. Therefore, a better understanding of IRGPs-based signatures may offer great potential for personalized management for LARC.

Cancer microenvironment and immune cell infiltration are reported to be correlated with cancer prognosis (30, 31), which was validated in our analysis. The related results showed that IPS can successfully differentiate between nCRT responses in LARCs, which demonstrated the reliability of this signature in assessing sensitivity to immunotherapy and nCRT. At the same time, the number of gene pairs composed of chemokine ligand C-X-C motif chemokine ligand 11 (CXCL11) was the largest in IPS, suggesting that the CXCL11 plays an important role in the immune scoring system. CXCL11 is involved in the progression of various cancers, and its expression is associated with tissue infiltration by T cells (32). Consistent with these findings, CXCL11 was identified as an independent prognostic biomarker in rectal cancer patients (33). Coincidentally, the gene pairs “CXCL11 > ZSCAN2” and “CXCL11 > LPR12” successfully differentiated CD8 T cell subtypes, which further once again underlines the mechanisms of CXCL11 in the immune response. Moreover, the type I anti-tumor T-cells have been proved to exhibit clinical relevance in various normal tissues and cancer types (26, 34), which were shown to correlate with the IPS-based immune score. Hence, our study shows that IPS can accurately distinguish the anti-tumor immuno-microenvironment from the irresponsive immuno-microenvironment in tumor tissues, and offered abundant clues for mechanisms and potential strategies to improve clinical treatment.

However, this study also has some limitations. The cohort of LARCs was equally dichotomized into high- and low-IPS groups, but, the substantial proportion of LARCs with an anti-tumor immune microenvironment was not the case. What’s more, the number of biopsies for sequencing and microarray analysis was small, potentially causing bias. Further studies are needed to confirm immune-nCRT relations and the mechanisms behind this. In addition, the discrimination accuracy of CD8+ T cell subsets needs to use the latest and authoritative algorithms and tools, such as ImmuCellAI (35), ImmuCellAI-mouse (36), etc.

In summary, by integrating expression from bulk and single-cell data, as well as clinical information from databases, our study identified the IPS-based immuno-score signature that can effectively predict the responses of LARCs to nCRT. Importantly, the signature could enhance the identification of LARCs who are likely to respond to immunotherapy, and may provide novel clues for mechanisms of immunotherapy.

## Data availability statement

The datasets presented in this study can be found in online repositories. The names of the repository/repositories and accession number(s) can be found in the article/**Supplementary Material**.

## Author contributions

ZX and YG: conceptualization. YG: funding acquisition and project administration. ZX, SY, and YG: methodology. YL, MH, HQ, WP, and ST: supervision. ZX: writing—original draft. ZX, SY, GH, and YG: writing—review and editing. All authors contributed to the article and approved the submitted version.

## Funding

This study was supported by the National Natural Science Foundation of China (NSFC, 82060618), the Key Research and Development Program of Jiangxi Province (20203BBGL73184), Doctoral Fund of First Affiliated Hospital of Gannan Medical University.

## Acknowledgments

The authors want to thank all the participants in the research.

## Conflict of interest

The authors declare that the research was conducted in the absence of any commercial or financial relationships that could be construed as a potential conflict of interest.

## Publisher’s note

All claims expressed in this article are solely those of the authors and do not necessarily represent those of their affiliated organizations, or those of the publisher, the editors and the reviewers. Any product that may be evaluated in this article, or claim that may be made by its manufacturer, is not guaranteed or endorsed by the publisher.

## Supplementary material

The Supplementary Material for this article can be foundonline at: https://www.frontiersin.org/articles/10.3389/fonc.2022.993726/full#supplementary-material
